# Ecto-Nucleotide Triphosphate Diphosphohydrolase-2 (NTPDase2) Deletion Increases Acetaminophen-Induced Hepatotoxicity

**DOI:** 10.3390/ijms21175998

**Published:** 2020-08-20

**Authors:** Linda Feldbrügge, Katrin Splith, Ines Kämmerer, Sandra Richter, Anna Riddermann, Santiago Andres Ortiz Galindo, Felix Krenzien, Tobias Müller, Eva Csizmadia, Johann Pratschke, Simon C. Robson, Moritz Schmelzle

**Affiliations:** 1 Department of Surgery, Campus Charité Mitte and Campus Virchow Klinikum, Charité—Universitätsmedizin, corporate member of Freie Universität Berlin, Humboldt-Universität zu Berlin, and Berlin Institute of Health, 10117 Berlin, Germany; katrin.splith@charite.de (K.S.); sandra.richter@charite.de (S.R.); anna.riddermann@charite.de (A.R.); santiago.ortiz-galindo@charite.de (S.A.O.G.); felix.krenzien@charite.de (F.K.); johann.pratschke@charite.de (J.P.); moritz.schmelzle@charite.de (M.S.); 2 Translational Centre for Regenerative Medicine (TRM), Universität Leipzig, 04103 Leipzig, Germany; ines_kaemmerer@gmx.de; 3 Division of Hepatology and Gastroenterology, Medical Department, Charité—Universitätsmedizin Berlin, corporate member of Freie Universität Berlin, Humboldt-Universität zu Berlin, and Berlin Institute of Health, 10117 Berlin, Germany; tobias.mueller@charite.de; 4 Departments of Medicine and Anesthesia, Beth Israel Deaconess Medical Center, Harvard Medical School, Harvard University, Boston, MA 02215, USA; ecsizmad@bidmc.harvard.edu (E.C.); srobson@bidmc.harvard.edu (S.C.R.)

**Keywords:** Entpd2, NTPDase2, purinergic signaling, APAP hepatotoxicity

## Abstract

Ecto-nucleotidase triphosphate diphosphohydrolase-2 (NTPDase2) is an ecto-enzyme that is expressed on portal fibroblasts in the liver that modulates P2 receptor signaling by regulating local concentrations of extracellular ATP and ADP. NTPDase2 has protective properties in liver fibrosis and may impact bile duct epithelial turnover. Here, we study the role of NTPDase2 in acute liver injury using an experimental model of acetaminophen (APAP) intoxication in mice with global deletion of NTPDase2. Acute liver toxicity was caused by administration of acetaminophen in wild type (WT) and NTPDase2-deficient (*Entpd2* null) mice. The extent of liver injury was compared by histology and serum alanine transaminase (ALT). Markers of inflammation, regeneration and fibrosis were determined by qPCR). We found that *Entpd2* expression is significantly upregulated after acetaminophen-induced hepatotoxicity. *Entpd2* null mice showed significantly more necrosis and higher serum ALT compared to WT. Hepatic expression of IL-6 and PDGF-B are higher in *Entpd2* null mice. Our data suggest inducible and protective roles of portal fibroblast-expressed NTPDase2 in acute necrotizing liver injury. Further studies should investigate the relevance of these purinergic pathways in hepatic periportal and sinusoidal biology as such advances in understanding might provide possible therapeutic targets.

## 1. Introduction

Acute liver failure afflicts patients with no pre-existing liver disease, and leads to death in up to 50% of cases [1]. The causes for acute liver failure vary between countries and regions, with acute viral infections (most frequently hepatitis B virus) and drug intoxication being most common.

*N*-acetyl-para-aminophenol (APAP), also known as acetaminophen or paracetamol, is a prescription-free analgesic that is one of the most widely used drugs in the world [2]. In several developed countries, intentional and non-intentional overdoses with this drug are the most common causes of acute liver failure [3]. APAP is metabolized in the liver, resulting in the production of several intermediary metabolites, one of which, *N*-Acetyl-p-benzochinonimin (NAPQI) is hepatotoxic and at high levels results in hepatic necrosis [4].

The murine model of APAP intoxication is one of the most widely used in vivo systems to evaluate etiology, predisposition, and treatments of acute liver injury. This experimental model has some mechanistic similarity to acute liver failure induced by APAP in the clinic [5] and was therefore chosen for this study.

Purinergic signaling has been implicated in many, if not all, pathophysiologic processes [6]. Purinergic signaling responses are mediated by extracellular nucleosides and nucleotides, e.g., adenine triphosphate (ATP), adenine diphosphate (ADP), or the nucleoside derivative adenosine, which are the specific receptors together with membrane-bound ecto-enzymes that regulate the concentrations of the messenger molecules and thereby modulate the downstream effects [7]. This pathway has been shown to play a role in liver inflammation, immune regulation, regeneration, and development of fibrosis [8,9,10].

The family members of the CD39 ecto-nucleotidases were shown to be pivotal in regulating purinergic responses in inflammatory states. The prototype member of this family CD39/Entpd1 has been the focus of study over the past decade. Deletion of *Entpd1* has also been studied in a model of APAP-induced acute liver injury [11,12]. Here, we observed increased mortality in *Cd39* null mice intoxicated with APAP with evidence for activation of P2X7 and the inflammasome in driving liver damage.

Another key ecto-enzyme that regulates extracellular nucleotide concentrations is ecto-nucleotide triphosphate diphosphohydrolase-2 (ENTPD2 or NTPDase2), the second member of the CD39 family of ecto-nucleotidases. This ecto-enzyme NTPDase2 is a preferential ecto-ATPase with different biological activity to CD39, which exerts both ecto-ATPase and -ADPase activities. CD39 is expressed by the vasculature and immune cells of the liver sinusoid unlike NTPDase2, which is expressed by portal fibroblasts in the liver and regulates the proliferation of bile duct cells [13].

We have previously shown that NTPDase2 has a protective role in limiting chronic liver injury and ameliorating fibrogenesis [14]. Here, we investigated the role of NTPDase2 in acute liver injury using the described model of APAP intoxication and using globally NTPDase2-deficient (*Entpd2* null) mice that we designed and generated. We show that there is accentuation of APAP-induced liver injury in NTPDase2 (*Entpd2* null mice), comparable to that seen in the *Entpd1* null studies. These studies have implications both for the understanding of the pathogenesis of APAP-induced hepatotoxicity as well as in translational therapeutics for acute liver injury.

## 2. Results

### 2.1. Hepatic Entpd2 Is Upregulated in Wild-Type (WT) Mice after APAP-Induced Acute Liver Injury

**Figure 1:**
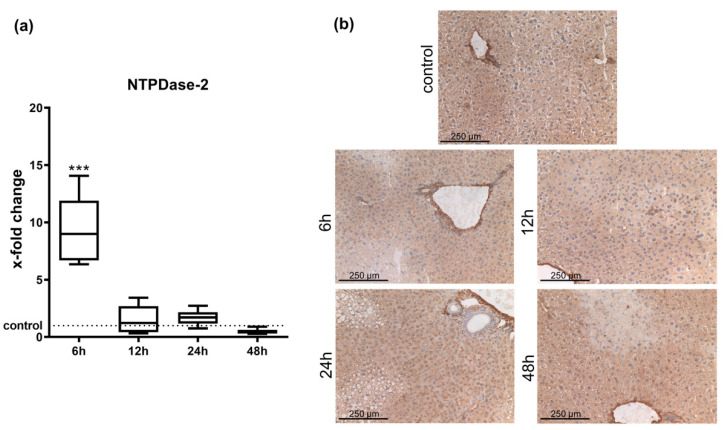
Ecto-nucleotidase triphosphate diphosphohydrolase-2 (NTPDase2) expression in wild type (WT) mice after acetaminophen (APAP) intoxication. (**a**) *Entpd2* expression by qPCR in WT whole liver tissue relative to vehicle treated controls, (**b**) immunohistochemistry for NTPDase2 in WT liver tissue of NTPDase2 (brown) at different time points after APAP intoxication (200×) *** *p* < 0.001. (*n* = 8 per time point).

Real-time qPCR of whole liver tissue after induction of acute liver failure by APAP injection revealed that WT mice showed significant increases in *Entpd2* expression at six hours when compared to animals treated with vehicle control (Figure 1a). After 12 h, mRNA levels were similar to controls.

The expression patterns of NTPDase2 in injured liver did not show any major changes in distribution after APAP induced acute liver injury when examined by immunohistochemistry. As in vehicle-treated control animals, expression of NTPDase2 in all cases was limited to periportal fibroblasts (Figure 1b).

### 2.2. Liver Necrosis after APAP Treatment Is Exacerbated in Entpd2 Null Mice

Two *Entpd2* null mice out of the group of eight animals, scheduled to be sacrificed at 48 h, were found dead in their cage on the morning of the day of euthanasia. All WT mice survived. This difference in survival was not statistically significant. At all other time points (6, 12, and 24 h after APAP), both WT and *Entpd2* null mice survived until the scheduled euthanasia and tissue harvest.

**Figure 2:**
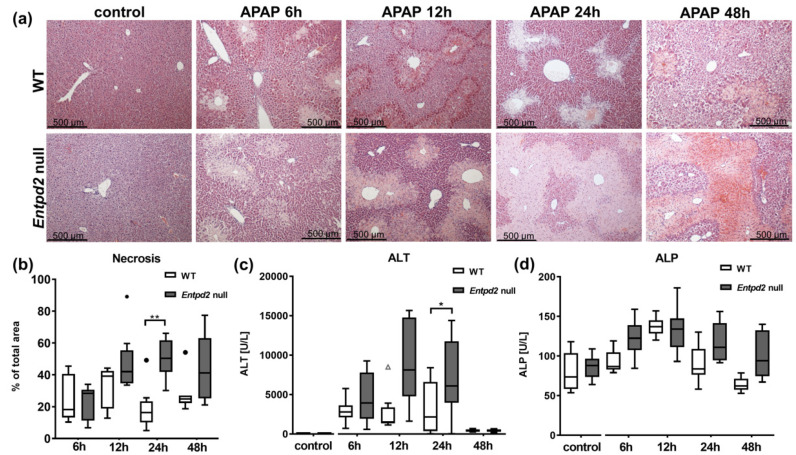
Liver injury after APAP intoxication in WT and *Entpd2* null mice. (**a**) Representative histopathological images of liver histology for controls and after 12 and 24 h post APAP intoxication by hematoxylin and eosin (H&E) staining in WT vs. *Entpd2* null livers (100×) (**b**). Percentage of necrotic areas per high-powered field (HPF), time course of (**c**) plasma ALT levels and (**d**) plasma ALP levels (*n* = 6–8 per time point). * *p* < 0.05, ** *p* < 0.01.

After induction of acute liver injury by injection of APAP, *Entpd2* null mice showed significantly more pronounced liver injury, as assessed by areas of necrosis on histological sections (Figure 2a). The increase in necrosis began to appear at 12 h after injection of APAP (WT 33% ± 13% vs. *Entpd2* null 48% ± 19%), which was statistically significant 24 h after APAP challenge (WT 19% ± 14% vs. *Entpd2* null 50% ± 11%, *p* = 0.003, Figure 2b). At 48 h after induction of acute liver injury, the area of necrosis did not increase further. At this time point, *Entpd2* null mice only displayed trends toward heightened levels of liver damage (WT 28% ± 11% vs. *Entpd2* null 44% ± 21%).

Concomitantly, elevation of serum alanine aminotransaminase (ALT) was significantly more pronounced in *Entpd2* null mice compared to WT mice (Figure 2c). This difference was statistically significant at 24 h after APAP intoxication; at 48 h, both WT and *Entpd2* null mice had similar and normalizing ALT levels, near those recorded in control animals. The same trend was observed in the measurements of the alkaline phosphatase (ALP) levels, even though no significance was found (Figure 2d).

### 2.3. Liver Regeneration and Proliferation of Liver Cells Are Not Enhanced in Entpd2 Null Mice

**Figure 3:**
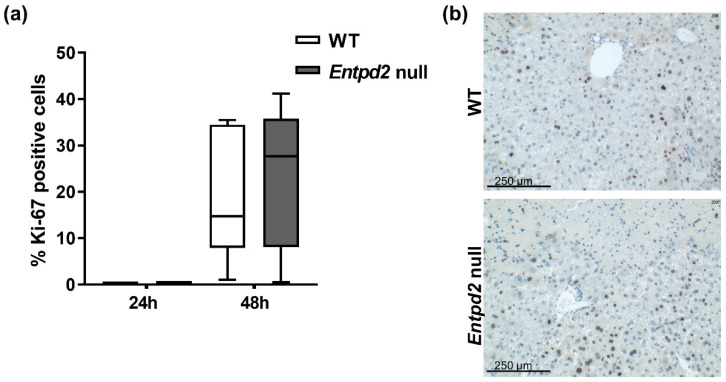
NTPDase2 deficiency does not impair liver cell proliferation after APAP intoxication. (**a**) Proliferation rate as percentage of Ki-67 positive cells out of all nucleated cells. (**b**) Representative images of Ki-67 staining in WT vs *Entpd2* null livers (200×), *n* ≥ 6 per genotype and time point.

We assessed the overall cellular proliferation rates in liver tissues by immunohistochemistry with tissues stained for the nuclear proliferation marker Ki-67. In healthy livers (controls) and at early time points after induction of acute liver injury (up to 24 h), hepatocytes appeared quiescent as regeneration became visible after this time. Forty-eight hours after injection of APAP, substantial proliferation of liver cells was visible (19% and 23% in WT and *Entpd2* null mice, respectively, Figure 3a). However, there were no significant differences between WT and *Entpd2* null mice after APAP.

The proliferating cells were chiefly hepatocytes. Cells of the periportal areas did not show substantial proliferation after APAP-induced acute liver injury, and the distribution of proliferating cells appeared similar in both WT and *Entpd2* null livers (Figure 3b).

### 2.4. Hepatic Expression of IL-6 and PDGF-B Is Upregulated in Entpd2 Null Mice after APAP Intoxication

**Figure 4:**
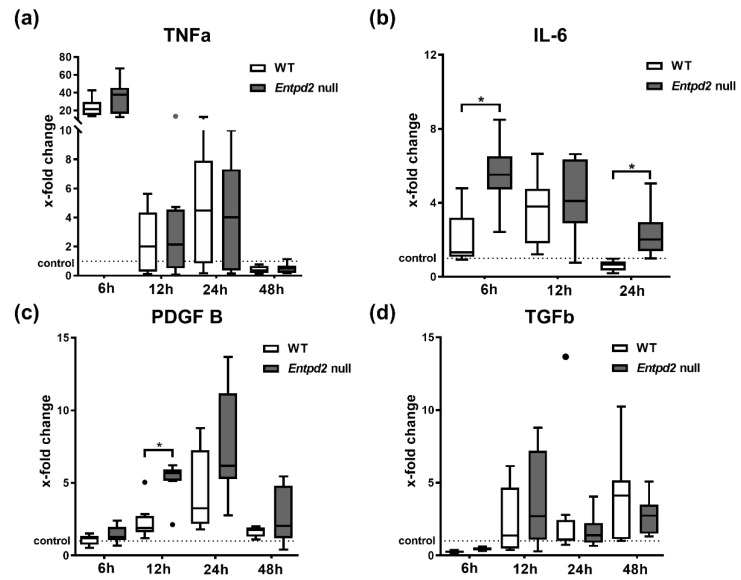
Hepatic expression of pro-inflammatory and pro-regenerative cytokines after APAP intoxication. qRT-PCR analysis of total mRNA isolated from mice livers after APAP intoxication. Data are shown as fold-changes of mRNA expression relative to vehicle-treated WT controls of (**a**) tumor necrosis factor (TNF-α), (**b**) interleukin-6 (IL-6), (**c**) platelet-derived growth factor-B (PDGF-B), and (**d**) transforming growth factor (TGF-β). * *p* < 0.05.

To explore potential mechanisms of heightened APAP induced liver injury in *Entpd2* null mice, we examined the expression of different markers that are linked to sterile liver inflammation, acute liver injury and liver regeneration: tumor-necrosis-factor-α (TNF-α), transforming growth factor-β (TGF-β), platelet derived growth factor-B (PDGF-B), and interleukin-6 (IL-6), in mouse liver tissue after APAP treatment. None of these analyzed markers exhibited significant differences in control WT and *Entpd2* null mice (data not shown). Six hours after APAP injection, TNF-α expression was upregulated in liver tissue, with no significant differences observed between WT and *Entpd2* null mice (Figure 4a). TNF-α mRNA expression then quickly decreased to around baseline levels after 12 h. IL-6 expression markedly increased in *Entpd2* null mice six hours after APAP treatment (31-fold (9–104) of control vs. 10-fold (3–36), *p* = 0.023), but is only significantly upregulated in WT mice after 12 h (Figure 4b). The increase in IL-6 mRNA remained detectable in *Entpd2* null mice for 24 h after the liver injury (12-fold increase (range of 7–22) at 24 h in *Entpd2* null), but this quickly decreased to baseline levels in WT mice. Similarly, the expression of PDGF-B was significantly higher in *Entpd2* null mice than in WT at 12 h after liver injury induction (2-fold (1.9–2.5) vs. 5-fold (2.5–10), *p* = 0.035). This parameter trended toward higher levels than in WT after 24 and 48 h, but without statistical significance at these later time points (Figure 4c). There was only a slight but significant increase in hepatic TGF-β mRNA starting 12 h after APAP injection, which remained elevated for at least 48 h. However, there were no difference between WT and *Entpd2* null mice (Figure 4d).

### 2.5. Hepatic Expression of Fibrosis-Associated Markers Is Triggered by APAP-Induced Liver Injury, Irrespective of Entpd2 Deletion

Repeated episodes of acute liver injury can provoke development of fibrosis, and we previously observed a protective role of NTPDase2 in fibrogenesis. To exclude the development of this in the acute setting in these mutant mice post-APAP, we also analyzed the established markers of fibrogenesis: vimentin, desmin, α-smooth muscle actin (α-SMA), and collagen.

**Figure 5:**
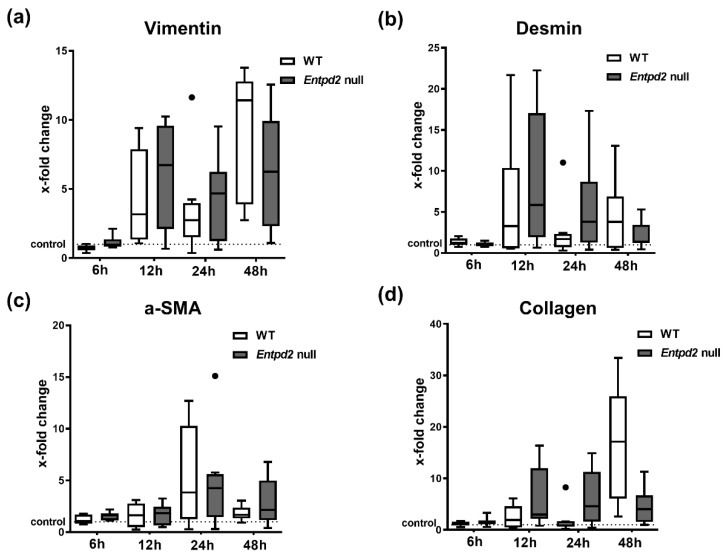
Hepatic expression of fibrosis-associated proteins after APAP intoxication. qRT-PCR analysis of total mRNA isolated from mice livers after APAP intoxication. Data are shown as fold-changes of mRNA expression relative to vehicle treated WT controls of (**a**) vimentin, (**b**) desmin, (**c**) smooth muscle actin (SMA), and (**d**) collagen.

We observed an increased expression of all these markers after APAP injection compared to vehicle-treated controls at varying time points. However, no differences between WT and *Entpd2* null mice was observed (Figure 5).

## 3. Discussion

In this current study, we have shown that *Entpd2* is upregulated in mice after APAP injury. More importantly, global deletion of NTPDase2 in mice worsens APAP-induced acute liver injury and increases liver necrosis. Our data suggest inducible and protective roles of this ecto-enzyme expressed on portal fibroblasts in acute necrotizing liver injury, which has not been previously described. This observation may be of importance in the understanding of APAP-induced liver injury.

We previously reported that NTPDase2 has hepatoprotective effects in that the gene deletion exacerbates CCl_4_ induced liver fibrosis [14]. The pathophysiology of this model of fibrosis, which takes several weeks to develop, is inherently different from the more rapid consequences of acute liver injury by APAP intoxication, which is visible within hours. The effect of NTPDase2 deletion on acute liver injury might therefore provide novel insights into NTPDase2 and other portal fibroblasts dependent regulatory and protective mechanisms in liver injury.

We previously showed that NTPDase1 (CD39) also displays protective effects in models of sclerosing cholangitis and biliary fibrosis [10,15]. CD39 plays a well-established role in thromboregulation and immune modulation, and is known to suppress T cells and macrophages by generation of immunosuppressive adenosine [16,17]. The protective effects of CD39 in liver fibrosis have therefore largely been ascribed to its role in immune regulation, supported by the observation of increased fibrosis after targeted deletion of CD39 in macrophages [10].

CD39 deletion has also been studied in a model of APAP-induced acute liver injury [11,12]. Here, we observed significantly increased mortality in *Cd39* null mice and elevated liver necrosis, as also shown here for *Entpd2* null mice. Unlike CD39, NTPDase2 is not expressed by endothelium or immune cells, making protective immune-regulatory mechanisms comparable to CD39 improbable [18].

NTPDase2 is expressed on periductal and periportal fibroblasts in the liver, where it was suggested to regulate the proliferation of bile duct cells by scavenging extracellular ATP and thus modulating P2Y signaling in ductular epithelial cells [13]. We observed a higher degree of liver injury in *Entpd2* null mice compared to WT mice, measured by histological necrosis and correlated by serum ALT levels. We also found a non-significant trend in increased requirement for euthanasia/mortality in *Entpd2* null mice when compared to WT. The heightened liver injury in knockout mice commenced as a trend at 12 h with important peaks at 24 h following APAP intoxication. In other models of inflammatory disease, NTPDase2 was suggested to indirectly modulate sterile inflammation by decreased local extracellular ATP hydrolysis, even though not directly expressed by immune cells [19,20,21].

We showed here that liver cells begin proliferating between 24 and 48 h after APAP administration, which is in line with other reports describing peak proliferation rates at 48–72 h [22,23]. At 48 h, however, we did not observe significant differences in either rate or pattern of proliferation of liver cells between WT and *Entpd2* null mice.

We therefore propose that the protective effects of NTPDase2, which is expressed in periportal areas and upregulated after APAP-induced liver injury, are mediated through scavenging local hepatotoxic ATP, released from necrotic liver cells and the preferential conversion to extracellular ADP. This is in agreement with our prior work in APAP [11,12] and the study by Amaral et al., who demonstrated both significant ATP release from necrotic cells after APAP treatment and direct ATP toxicity through increased intracellular Ca^2+^ signaling [24]. We agree with these authors that extracellular ATP toxicity is mediated through P2 receptor signaling and could be, at least in part, abrogated by apyrase (soluble NTPDase).

The complexity of the pathophysiology of APAP-induced acute liver injury is underlined by the controversy with which some of these pathways were discussed in the literature: Jaeschke et al. examined and reviewed mechanisms of APAP-induced acute liver injury, and focused on intracellular processes caused by APAP metabolites. These include the formation of free radicals that directly cause hepatocyte necrosis [25]. The protective effects of a purinergic receptor antagonist A438079, which results in inhibition of P2X7 receptors and dampens the inflammasome activation in APAP injury [11], were also attributed to direct inhibition of hepatic P450 enzyme activity [26]. However, there are also direct toxic effects on hepatocytes by extracellular ATP [24], which substantiate the findings in earlier studies that focused on mechanistic roles for CD39 and P2X7 in the mediation of immune cells and inflammation in APAP-induced liver injury.

We showed upregulation of TNF-α, TGF-β, IL-6, and PDGF-B in liver tissues within 48 h following APAP administration. IL-6 and PDGF-B were significantly higher in *Entdp2* null mice compared to WT. Whereas TNF-α is most commonly viewed as a pro-inflammatory agent in acute liver failure [27,28], IL-6 can directly protect hepatocytes from oxidative stress after APAP toxicity, and both have long been known to stimulate liver regeneration [29,30]. PDGF-B and TGF-β are secreted by damaged hepatocytes after toxic insults and can stimulate regeneration, but can also trigger induction of fibrosis [31]. The elevation of IL-6 and PDGF-B in *Entpd2* null mice early in APAP-induced liver acute injury can be seen as another indicator of the mitigating effects of NTPDase2 in the initial necrotizing phase of liver injury.

These data might already indicate a higher susceptibility to liver fibrosis, which we observed after CCl_4_ treatment, which also induces substantial tissue necrosis initially [14]. Other markers of fibrogenesis, such as collagen, vimentin, desmin, and α-SMA, are up-regulated after APAP administration, but with more delay than the mentioned cytokines and without significant difference between the genotypes. We considered, at these early time points, at the relatively low dose of 300 mg/kg, and in the absence of repetitive dosing, that no relevant fibrosis was observed [23].

Taken together, our findings demonstrate a protective role of NTPDase2 and portal fibroblasts in the context of acute APAP injury. Further studies should investigate the relevance of these purinergic pathways and portal fibroblast functionality to human liver disease as these might provide future therapeutic targets.

## 4. Materials and Methods

### 4.1. Animals

The generation of global NTPDase2 deficient (*Entpd2* null) mice on a C57BL/6 background has been described previously [32,33]. For all experiments, we used 7- to 9-week-old male mice weighing 20–27 g with weight-, age-, sex-, and strain-matched WT mice as controls (Charles River, Sulzfeld, Germany). The animal experiment protocol was reviewed and approved (4 March 2013) by the respective regional government agency of the state of Saxony, Germany (TVV 49/12) and was performed according to international guidelines on the use of laboratory animals.

### 4.2. Induction of Acute Liver Injury by APAP

Animals were injected intraperitoneally with 300 mg/kg of acetaminophen (APAP, Sigma-Aldrich, Taufkirchen, Germany) after 16 h of starvation. APAP solution was made freshly for each experiment in warm phosphate-buffered saline (PBS) (50 °C) at 15 mg/mL and injected after cooling to mouse body temperature. Control animals were injected with the same volume of phosphate-buffered saline (PBS). Mice were euthanized using a mixture of atropine (0.1 mg/kg), ketamine (100 mg/kg), and xylazine (5 mg/kg) at 6, 12, 24, or 48 h after APAP or vehicle injection for the collection of blood and liver tissue. At each time point, eight animals were treated with APAP and two animals were treated with vehicle as controls. For statistical analysis, control animals of all time points were combined into one group per genotype.

### 4.3. Measurement of Serum ALT and ALP

Blood was drawn into syringes containing citrate phosphate dextrose (CPD) solution as anticoagulant. Plasma was obtained by centrifugation at 1000× *g* for 10 min, snap frozen in liquid nitrogen and stored at –80 °C until further use. Alanine aminotransferase (ALT) and alkaline phosphatase (ALP) activities were measured in plasma using Reflotron^®^ sticks with the Reflotron^®^ Plus system (Roche Diagnostics, Mannheim, Germany).

### 4.4. RNA Isolation and Quantitative Real Time Polymerase Chain Reaction (qPCR)

Liver tissue was snap frozen in liquid nitrogen after harvest and stored at –80 °C until further processing. The tissue was homogenized with QIAzol^®^ Lysis Reagent (QIAGEN, Hilden Germany) to isolate RNA according to the manufacturer’s protocol and 1 µg of RNA was transcribed to cDNA using the RevertAid First Strand cDNA Synthesis Kit, (Life Technologies, Karlsruhe, Germany), which contains both oligo (dT)_18_ and random hexamer primers.

qPCR was performed on a 7500 Real-Time PCR System (Applied Biosystems by Life Technologies, Carlsbad, CA, USA) using standard protocols and the GoTaq qPCR Master Mix (Promega, Mannheim, Germany). The following *Entpd2* primers were used as published previously [8]: Fw: 5′-TGACTGCCAACTACCTGCTG-3′, Rev: 5′-CCGCAAATGGACCTCATTAT-3′. Other primers were: *desmin*: Fw: 5′-CAGAGGCTCAAGGCCAAACTA-3′, Rev: 5′-GAACGCGATCTCCTCGTTGA-3′, *vimentin*: Fw: 5′-GCAGTATGAAAGCGTGGCTG-3′, Rev: 5′-CAGGGACTCGTTAGTGCCTTT-3′, *collagen*: Fw: 5′-GAGAGGTGAACAAGGTCCCG-3′, Rev: 5′-AAACCTCTCTCGCCTCTTGC-3′, *PDGF-B*: Fw: 5′-GGAGTCGGCATGAATCGCTG-3′, Rev: 5′-AATGGGATCCCCCTCGGC-3′, *TGF-β*: Fw: 5′-CTGCTGACCCCCACTGATAC-3′, Rev: 5′-AGCCCTGTATTCCGTCTCCT-3′, *TNF-α*: Fw: 5′-GGCCTCCCTCTCATCAGTTC-3′, Rev: 5′-CTCCACTTGGTGGTTTGCTAC-3′, *α-SMA:* Fw: 5′-TCCAGCTATGTGTGAAGAGG-3′, Rev: 5′-GCCAGATCTTTTCCATGTCG-3′, *GAPDH:* Fw: 5′-AGCTCATTTCCTGGTATGACA-3′; Rev: 5′-CTCTCTTGCTCAGTGTCCTT-3′. *IL-6* and *GAPDH:* gene expression assays Mm00446190_m1, Mm99999915_g1 (Applied Biosystems).

### 4.5. Immunohistochemistry

Livers were excised, and small sections were fixed in 10% formalin or snap frozen for histological analysis. Formalin-fixed, paraffin-embedded, or frozen murine liver tissues were cut into 6 µm sections. One slide from each block was stained by hematoxylin and eosin for morphological analysis. For immunohistochemistry, sections were fixed in acetone and blocked with 7% horse serum (Vector Labs, Burlingame, CA, USA) for half an hour. The tissues were first incubated with primary antibodies overnight at 4 °C (NTPDase2) or for 2 h at 37 °C (Ki-67). Primary antibodies were rabbit polyclonal anti-mouse NTPDase2 (http://ectonucleotidases-ab.com, as characterized previously [34] and Ki-67 (Dako, Santa Clara, CA, USA); both antibodies were used in 1:500 dilutions. After peroxidase and biotin activity blocking, sections were incubated with the biotinylated secondary antibody for one hour (goat polyclonal anti-rabbit IgG, Vector Labs), continued with Avidin-Biotin complex horseradish peroxidase (HRP) and visualized with ImmPACT DAB (Vector Labs). All slides were mounted on cytoseal, and examined and recorded on a Nikon microscope.

### 4.6. Statistical Analysis

All results are reported as mean ± standard deviation (SD), box-and-whisker plots show the median. The statistical analyses were performed with GraphPad Prism v8.4.3 (GraphPad Software, San Diego, CA, USA). Two-tailed Student’s *t* test or Mann–Whitney rank sum test as well as ANOVA or Kruskal–Wallis test were used to test for significant differences between two or more groups. Normal distribution was tested via Shapiro–Wilk test. A *p* value of <0.05 was considered significant.

## References

[1] Bernal W., Lee W.M., Wendon J., Larsen F.S., Williams R. (2015). Acute liver failure: A curable disease by 2024?. J. Hepatol..

[2] Aminoshariae A., Khan A. (2015). Acetaminophen: Old Drug, New Issues. J. Endod..

[3] Bernal W., Wendon J. (2013). Acute Liver Failure. N. Engl. J. Med..

[4] Yoon E., Babar A., Choudhary M., Kutner M., Pyrsopoulos N. (2016). Acetaminophen-Induced Hepatotoxicity: A Comprehensive Update. J. Clin. Transl. Hepatol..

[5] Maes M., Vinken M., Jaeschke H. (2016). Experimental models of hepatotoxicity related to acute liver failure. Toxicol. Appl. Pharmacol..

[6] Eltzschig H.K., Sitkovsky M.V., Robson S.C. (2012). Purinergic Signaling during Inflammation. N. Engl. J. Med..

[7] Burnstock G. (2012). Purinergic signalling: Its unpopular beginning, its acceptance and its exciting future. BioEssays.

[8] Beldi G., Wu Y., Sun X., Imai M., Enjyoji K., Csizmadia E., Candinas D., Erb L., Robson S.C. (2008). Regulated Catalysis of Extracellular Nucleotides by Vascular CD39/ENTPD1 Is Required for Liver Regeneration. Gastroenterology.

[9] Yoshida O., Kimura S., Jackson E.K., Robson S.C., Geller D.A., Murase N., Thomson A.W. (2013). CD39 expression by hepatic myeloid dendritic cells attenuates inflammation in liver transplant ischemia-reperfusion injury in mice. Hepatology.

[10] Rothweiler S., Feldbrügge L., Jiang Z.G., Csizmadia E., Longhi M.S., Vaid K., Enjyoji K., Popov Y.V., Robson S.C. (2019). Selective deletion of ENTPD1/CD39 in macrophages exacerbates biliary fibrosis in a mouse model of sclerosing cholangitis. Purinergic Signal..

[11] Hoque R., Sohail M.A., Salhanick S., Malik A.F., Ghani A., Robson S.C., Mehal W.Z. (2012). P2X7 receptor-mediated purinergic signaling promotes liver injury in acetaminophen hepatotoxicity in mice. Am. J. Physiol. Gastrointest. Liver Physiol..

[12] Schmelzle M., Splith K., Andersen L.W., Kornek M., Schuppan D., Jones-Bamman C., Nowak M., Toxavidis V., Salhanick S.D., Han L. (2013). Increased plasma levels of microparticles expressing CD39 and CD133 in acute liver injury. Transplantation.

[13] Jhandier M.N., Kruglov E.A., Lavoie E.G., Sévigny J., Dranoff J.A. (2005). Portal fibroblasts regulate the proliferation of bile duct epithelia via expression of NTPDase2. J. Biol. Chem..

[14] Feldbrügge L., Jiang Z.G., Csizmadia E., Mitsuhashi S., Tran S., Yee E.U., Rothweiler S., Vaid K.A., Sévigny J., Schmelzle M. (2018). Distinct roles of ecto-nucleoside triphosphate diphosphohydrolase-2 (NTPDase2) in liver regeneration and fibrosis. Purinergic Signal..

[15] Peng Z.W., Rothweiler S., Wei G., Ikenaga N., Liu S.B., Sverdlov D.Y., Vaid K.A., Longhi M.S., Kuang M., Robson S.C. (2017). The ectonucleotidase ENTPD1/CD39 limits biliary injury and fibrosis in mouse models of sclerosing cholangitis. Hepatol. Commun..

[16] Deaglio S., Dwyer K.M., Gao W., Friedman D., Usheva A., Erat A., Chen J.F., Enjyoji K., Linden J., Oukka M. (2007). Adenosine generation catalyzed by CD39 and CD73 expressed on regulatory T cells mediates immune suppression. J. Exp. Med..

[17] Allard B., Longhi M.S., Robson S.C., Stagg J. (2017). The ectonucleotidases CD39 and CD73: Novel checkpoint inhibitor targets. Immunol. Rev..

[18] Dranoff J.A., Kruglov E.A., Robson S.C., Braun N., Zimmermann H., Sévigny J. (2002). The ecto-nucleoside triphosphate diphosphohydrolase NTPDase2/CD39L1 is expressed in a novel functional compartment within the liver. Hepatology.

[19] Feldbrügge L., Moss A.C., Yee E.U., Csizmadia E., Mitsuhashi S., Longhi M.S., Sandhu B., Stephan H., Wu Y., Cheifetz A.S. (2017). Expression of Ecto-nucleoside Triphosphate Diphosphohydrolases-2 and -3 in the Enteric Nervous System Affects Inflammation in Experimental Colitis and Crohn’s Disease. J. Crohns Colitis.

[20] Grubišić V., Perez-Medina A.L., Fried D.E., Sévigny J., Robson S.C., Galligan J.J., Gulbransen B.D. (2019). NTPDase1 and -2 are expressed by distinct cellular compartments in the mouse colon and differentially impact colonic physiology and function after DSS colitis. Am. J. Physiol. Gastrointest. Liver Physiol..

[21] Liñán-Rico A., Turco F., Ochoa-Cortes F., Harzman A., Needleman B.J., Arsenescu R., Abdel-Rasoul M., Fadda P., Grants I., Whitaker E. (2016). Molecular Signaling and Dysfunction of the Human Reactive Enteric Glial Cell Phenotype: Implications for GI infection, IBD, POI, Neurological, Motility and GI Disorders. Inflamm. Bowel Dis..

[22] James L.P., Kurten R.C., Lamps L.W., McCullough S., Hinson J.A. (2005). Tumour Necrosis Factor Receptor 1 and Hepatocyte Regeneration in Acetaminophen Toxicity: A Kinetic Study of Proliferating Cell Nuclear Antigen and Cytokine Expression. Basic Clin. Pharmacol. Toxicol..

[23] Bhushan B., Walesky C., Manley M., Gallagher T., Borude P., Edwards G., Monga S.P.S., Apte U. (2014). Pro-Regenerative Signaling after Acetaminophen-Induced Acute Liver Injury in Mice Identified Using a Novel Incremental Dose Model. Am. J. Pathol..

[24] Amaral S.S., Oliveira A.G., Marques P.E., Quintão J.L., Pires D.A., Resende R.R., Sousa B.R., Melgaço J.G., Pinto M.A., Russo R.C. (2013). Altered responsiveness to extracellular ATP enhances acetaminophen hepatotoxicity. Cell Commun. Signal..

[25] Jaeschke H., Ramachandran A. (2020). Mechanisms and pathophysiological significance of sterile inflammation during acetaminophen hepatotoxicity. Food Chem. Toxicol..

[26] Xie Y., Williams C.D., McGill M.R., Lebofsky M., Ramachandran A., Jaeschke H. (2013). Purinergic receptor antagonist A438079 protects against acetaminophen-induced liver injury by inhibiting p450 isoenzymes, not by inflammasome activation. Toxicol. Sci..

[27] Xu H., Li H., Cao D., Wu Y., Chen Y. (2014). Tumor necrosis factor α (TNF-α) receptor-I is required for TNF-α-mediated fulminant virus hepatitis caused by murine hepatitis virus strain-3 infection. Immunol. Lett..

[28] Chastre A., Bélanger M., Beauchesne E., Nguyen B.N., Desjardins P., Butterworth R.F. (2012). Inflammatory cascades driven by tumor necrosis factor-alpha play a major role in the progression of acute liver failure and its neurological complications. PLoS ONE.

[29] Gao R.Y., Wang M., Liu Q., Feng D., Wen Y., Xia Y., Colgan S.P., Eltzschig H.K., Ju C. (2020). Hypoxia-Inducible Factor-2α Reprograms Liver Macrophages to Protect Against Acute Liver Injury Through the Production of Interleukin-6. Hepatology.

[30] Li S.Q., Zhu S., Han H.M., Lu H.J., Meng H.Y. (2015). IL-6 trans-signaling plays important protective roles in acute liver injury induced by acetaminophen in mice. J. Biochem. Mol. Toxicol..

[31] Mekala S., Tulimilli S.V., Geesala R., Manupati K., Dhoke N.R., Das A. (2018). Cellular crosstalk mediated by platelet-derived growth factor BB and transforming growth factor β during hepatic injury activates hepatic stellate cells. Can. J. Physiol. Pharmacol..

[32] Gampe K., Stefani J., Hammer K., Brendel P., Pötzsch A., Enikolopov G., Enjyoji K., Acker-Palmer A., Robson S.C., Zimmermann H. (2014). NTPDase2 and Purinergic Signaling Control Progenitor Cell Proliferation in Neurogenic Niches of the Adult Mouse Brain. Stem Cells.

[33] Vandenbeuch A., Anderson C.B., Parnes J., Enjyoji K., Robson S.C., Finger T.E., Kinnamon S.C. (2013). Role of the ectonucleotidase NTPDase2 in taste bud function. Proc. Natl. Acad. Sci. USA.

[34] Bartel D.L., Sullivan S.L., Lavoie E.G., Sevigny J., Finger T.E. (2006). Nucleoside triphosphate diphosphohydrolase-2 is the ecto-ATPase of type I cells in taste buds. J. Comp. Neurol..

